# Oxygen Sensing of Pt/PEO-TiO_2_ in Humid Atmospheres at Moderate Temperatures

**DOI:** 10.3390/s21072558

**Published:** 2021-04-06

**Authors:** Bernd Engelkamp, Klaus Schierbaum

**Affiliations:** Abteilung für Materialwissenschaft, Institut für Experimentelle Physik der Kondensierten Materie, Heinrich-Heine-Universität Düsseldorf, Universitätsstraße 1, 40225 Düsseldorf, Germany; klaus.schierbaum@hhu.de

**Keywords:** plasma electrolytic oxidation, titanium dioxide, Pt/TiO_2_, ionosorption, oxygen gas sensor

## Abstract

Here, we show that the presence of adsorbed water improves the oxygen-sensing properties of Pt/TiO${}_{2}$ at moderate temperatures. The studied interface is based on porous plasma electrolytic oxidized titanium (PEO-TiO${}_{2}$) covered with platinum clusters. The electrical resistance across Pt/PEO-TiO${}_{2}$ is explained by an electronic depletion layer. Oxygen adsorbates further increase the depletion by inducing extrinsic interface states, which are occupied by TiO${}_{2}$ conduction band electrons. The high oxygen partial pressure in ambient air substantially limits the electron transport across the interface. Our DC measurements at defined levels of humidity at 30 ${}^{\circ }$C show that adsorbed water counteracts this shortcoming, allowing oxygen sensing at room conditions. In addition, response and recovery times from temporal oxygen exposure decrease with humidity. We attribute the effects to competing adsorption processes and reactions of water with adsorbed oxygen species and/or lattice oxygen, which involve electron re-injection to the TiO${}_{2}$ conduction band. Elevated temperatures up to 170 ${}^{\circ }C$ attenuate the effects, presumably due to the lower binding strength to the surface of molecular water compared with oxygen adsorbates.

## 1. Introduction

Titanium dioxide is commonly investigated as gas sensing material [1]. For this purpose, TiO${}_{2}$ is mainly used in chemoresistive gas sensors, where electrical properties are determined as functions of the ambient gas [2]. The performance highly depends on the morphology of the gas sensing material. In general, porous TiO${}_{2}$ with a large surface-to-volume ratio provides many active sites for adsorption, which is essential for high sensitivity [3,4]. In this respect, plasma electrolytic oxidized titanium (PEO-TiO${}_{2}$) with high porosity was already proved to be suitable for the detection of H${}_{2}$, H${}_{2}$O and CO [5].

The conceptual setup follows a sandwich structure with a permeable top electrode, PEO-TiO${}_{2}$ and titanium substrate as the bottom electrode. The top electrode often contains platinum as a catalytically active metal. Due to work function differences between Pt and the n-type TiO${}_{2}$, an electronic depletion within the TiO${}_{2}$ is formed. The charge distribution induces band bending, which represents a rectifying resistance for electronic charge transport across the interface. Analogously, chemisorbed gases at three phase boundaries (TPBs), i.e., gas/Pt/TiO${}_{2}$, affect the depleted layer [6]. In case of oxidzing adsorbates, conduction band electrons from TiO${}_{2}$ are localized in extrinsic surface states. Therefore, the depletion in TiO${}_{2}$ increases and ultimately controls the interface resistance. The fundamental mechanism is often referred to as acceptor-type chemisorption or ionosorption [6,7]. Herein, the most prominent acceptor gas in ambient air is oxygen. The net reaction

(1) $$O_{2}+V_{\mathrm{ads}}+e^{-}⇌O_{2\;\mathrm{ads}}^{-}$$

indicates the ionosorption of molecular oxygen with $O_{2\;\mathrm{ads}}^{-}$ as the ionosorbed molecule; $V_{\mathrm{ads}}$ represents a free adsorption site at an undercoordinated surface titanium atom. By dissociation and further electron acceptance, an atomic species forms according to the reaction

(2) $$O_{2\;\mathrm{ads}}^{-}+V_{\mathrm{ads}}+e^{-}⇌2{O^{-}}_{\mathrm{ads}}.$$

The nature of the ionosorbed species is assumed to depend on the adsorption temperature and the substrate [7]. A dissociative adsorption of molecular oxygen at catalytically active platinum and the subsequent spill-over effect to TiO${}_{2}$ promote the accumulation of atomic ionosorbed oxygen [6]. In sum, the surface density of ionosorbed oxygen controls the major contribution to the band bending. One approach to exploit this effect for oxygen sensing is to apply a forward voltage, which decreases the band bending and promotes an electronic current across the Pt/TiO${}_{2}$ interface. Due to the exponential I–V relation, a highly sensitive current signal in dependence of the oxygen adsorbates is expected.

In general, the effect is conceivable for the operation of a sensor at room temperature. This is in contrast with many conventional chemoresistive sensors based on semiconducting metal oxides, where the change in resistance is mainly caused by the alteration in lattice point-defect concentrations. Therefore, high operating temperatures are essential to attain defect equilibriums at appropriate time scales [8]. At low temperatures (e.g., below 200 ${}^{\circ }C$), the dynamics of lattice point defects can be neglected and the change in resistance is mainly attributed to the effect of reactive adsorbates. For instance, an early study at low pressures showed that the Pt/TiO${}_{2}$(110) interface is sensitive to low partial pressures of oxygen (e.g., Δp = $10^{-3}$ Pa) at 130 ${}^{\circ }C$ [9].

However, there are two major problems to overcome to use the effect for oxygen gas sensors. First, high oxygen partial pressures are problematic. At room atmosphere, the surface is almost saturated with adsorbed oxygen. Thus, the high surface density of ionosorbed oxygen prevents almost any current across the Pt/TiO${}_{2}$ interface. In addition, small deviations in the relatively high oxygen partial pressure have a rather small impact on the band bending. Second, the cross-selectivity to other reactive gases must be respected. Water vapor has presumably the most prominent role in ambient air. Even in vacuum experiments, water adsorption from residual water on defective TiO${}_{2}$ is inevitable. Contrary to the effect of oxygen adsorbates, adsorbed water showed an electron donor effect on semiconducting metal oxides [10]. Although it is frequently used for humidity sensors, a non-constant concentration of adsorbed water interferes with oxygen detection [11,12]. In addition, the direct interaction of adsorbed oxygen species and water molecules or water derived species represents an additional step in complexity [13].

However, we show that oxygen detection with Pt/PEO-TiO${}_{2}$ can also benefit from the presence of water. Therefore, we investigated the current across the Pt/PEO-TiO${}_{2}$ interface as a function of the oxygen concentration and relative humidity. From the results, the reactions of oxygen and water on the surface are considered.

## 2. Materials and Methods

PEO preparation is extensively described and analyzed in previous studies [14,15] and is only briefly explained here. Titanium samples with 125 μm thickness were plasma electrolytic oxidized in 75 wt % sulphuric acid. The galvanostatic operation mode is limited to 55 mA/cm${}^{2}$ and is stopped at a charge density of approximately 42 C/cm${}^{2}$, which roughly corresponds to 145 V. After treatment, the sample was rinsed in deionized water and dried in air.

X-ray photoelectron spectra were recorded with Al Kα radiation with a beam diameter around 100 μm and energy resolution of 0.5 eV (PHI 5000 VersaProbe II, Physical Electronics Inc., Chanhassen, MN, USA). The residual pressure was about $10^{-8}$ mbar. Binding energies refer to the C 1s peak at 284.8 eV. For the deconvolution of the O 1s core level peak a multi peak fit with “Voigt” profiles and a “Tougaard” background was used. Water adsorption/desorption isotherms of PEO-TiO${}_{2}$ were recorded with a humidity-controlled thermobalance (Q5000 SA, TA instruments Inc., New Castle, DE, USA) with time intervals of 40 min and 5% steps in relative humidity (r.h.) Before the measuring, the PEO-TiO${}_{2}$ was mechanically separated from the titanium substrate.

A single sample for the present study had an area of 3 × 3 mm${}^{2}$. Two platinum areas of 1.4 mm${}^{2}$ and approximately 100 nm thickness were sputtered onto PEO-TiO${}_{2}$ at room temperature. The spacing between the two areas was 0.6 mm. Underneath one of them, the TiO${}_{2}$ surface was scratched before sputtering to obtain an ohmic contact to the titanium. Electrical contacts were realized by gold-wire bonding to the platinum areas. The operating temperature was regulated by means of a platinum microheater directly below the sensor material and an RTD Pt1000. The electrical current was measured with a PXI-4071 DMM (National Instruments Corp., Austin, TX, USA). The resolution in DC current operation was below 10 nA in the measurement range of 10 mA.

The gas sensing experiments were performed in a gas-flow apparatus. Nitrogen 5.0 and oxygen 6.0 functioned as the inert carrier gas and test gas, respectively. Nitrogen was additionally purified by oxygen and humidity gas filters (CP17970 and CP17971, Varian Inc., Palo Alto, CA, USA). Two mass flow controllers (MFCs, F-201CV, Bronkhorst Deutschland Nord GmbH, Kamen, Germany) regulated the ratio between O${}_{2}$ and N${}_{2}$. An equivalent MFC was used to adjust the flow of water vapor saturated N${}_{2}$, corresponding to 100% r.h. The total flow was kept constant at 100 mL/min. The investigation of different levels of r.h. started in a dry nitrogen flow. Initially, the sample was heated for one hour to approximately 120 ${}^{\circ }C$ to decrease the amount of adsorbed water. Before each measurement, a minimum time interval of 10 h at a constant level of humidity was applied to ensure a quasi-equilibrium with the water vapor. The microstructure of the platinum covered oxide layer was investigated by field emission scanning electron microscopy (SEM; JSM-7500F, JEOL Ltd., Tokio, Japan).

## 3. Results and Discussion

**Figure 1:**
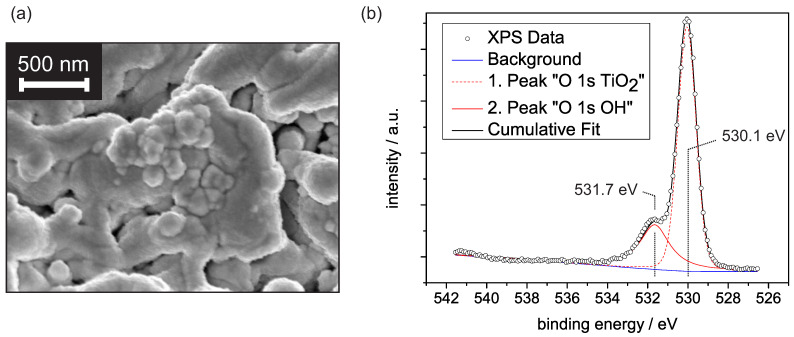
(**a**) SEM image of the Pt/PEO-TiO${}_{2}$ surface. (**b**) XPS spectra of the O 1s binding energy range for the PEO-TiO${}_{2}$ sample at room temperature. The first peak is centered around 530.1 eV and is assigned to O${}^{2-}$, while the second peak is centered around 531.7 eV and is assumed to originate from OH groups.

Figure 1a shows an electron microscope image of the porous PEO-titanium covered by platinum. The porosity with distinct fissures and unevenly distributed, inhomogeneous pores is apparent. The rough surface texture of the structure originates from the platinum coverage and indicates the formation of platinum clusters. Previous XRD investigations revealed a dominant rutile fraction of 93.7%, with the (110) surface showing the highest intensity contribution [15]. This dominance is presumably explained by the thermodynamically lowest surface energy compared to other rutile surfaces [16]. For this reason, we refer in the following discussion to some concepts from the well-researched TiO${}_{2}$ rutile (110) surface.

### 3.1. Water Adsorption on PEO-TiO${}_{2}$

Due to the relevance of humidity effects on metal oxide-based gas sensors, we first focus on the water adsorption on PEO-TiO${}_{2}$ at constant levels of relative humidity (r.h.) Although a weak external electrical field is generated by the applied voltage in our experimental setup, we did not expect any noticeable effect on the water adsorption behavior, since at the given magnitude, the intrinsic field largely controls the adsorption [17]. It is generally accepted that the water adsorption on TiO${}_{2}$ can be described within the concept of multilayer adsorption [11,18,19]. Basic requisite for multilayer adsorption is the formation of an innermost layer. This layer strongly depends on the molecular structure of the TiO${}_{2}$ surface and defines whether a hydrophobic or hydrophilic surface is formed [20]. Typically, the innermost layer consists of hydroxyl groups formed by water dissociation, although molecular water with rigid mobility may occur [11,19]. Previous studies showed that dissociative adsorption is highly promoted by surface defects and steps [21]. Given the PEO-TiO${}_{2}$ surface, with its large density of defects and steps, we assumed an initial dissociative water adsorption to occur even at room temperature. Figure 1b shows a spectra including the O 1s binding energies determined with X-ray photoelectron spectroscopy on PEO-TiO${}_{2}$ at room temperature. The deconvolution of the data reveals two peaks. The first peak is assigned to O${}^{2-}$, i.e., mainly lattice oxygen, while the second peak origins from OH groups [22]. A third peak due to H_2_O adsorbates would be expected around 532.5–532.7 eV and can not be determined in our spectra [22]. This qualitatively confirms that OH groups predominate over H_2_O adsorbates at around $10^{-8}$ mbar.

After formation of the hydroxyle monolayer on PEO-TiO${}_{2}$, water molecules adsorb physically via hydrogen bonds at surface hydroxyl groups and form the first physisorbed water layer [11,18,19,23]. The bond between surface hydroxyl groups and physisorbed water molecules is stronger compared with the water–water interaction in the following layers of water, especially when single water molecules bind to two surface hydroxyl groups [23,24]. When the first water layer is completed, further water molecules bind via hydrogen bonds (physisorbed water). The bond strength between water molecules decreases with increasing distance from the surface and the structure progressively resembles liquid water [11].

**Figure 2:**
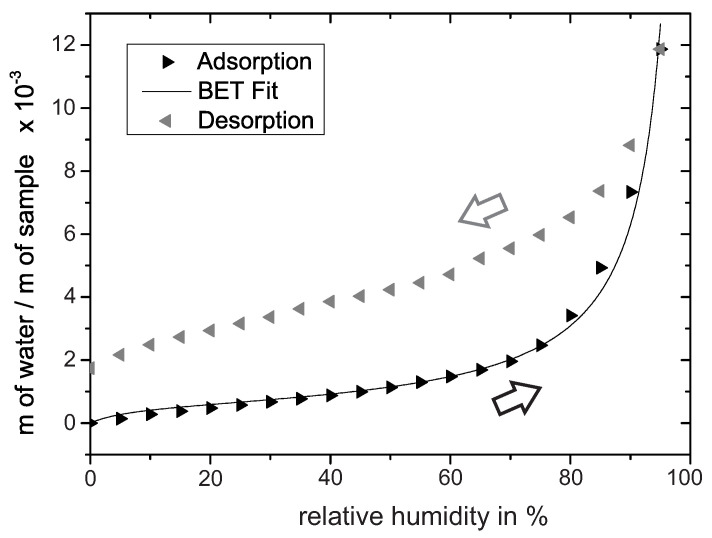
Water adsorption/desorption isotherm recorded at 30 ${}^{\circ }C$ reveals insights into the water adsorption process on PEO-TiO${}_{2}$. The adsorption isotherm is additionally fitted by the BET equation.

To confirm the formation of multilayers on PEO-TiO${}_{2}$, water adsorption/desorption isotherms at 30 ${}^{\circ }C$ were recorded with a humidity-controlled thermobalance. Prior to the experiment, the oxide was heated to 120 ${}^{\circ }C$ for 1 h to reduce the surface concentration of water adsorption species. The adsorption isotherm in Figure 2 shows first an increase in slope around 0–20% r.h. In the concept of BET-multilayer adsorption (BET: Brunauer–Emmett–Teller), the subsequent inflection point often indicates the transition from monolayer adsorption to multilayer adsorption [25]. By fitting the BET equation to the experimental data, we determined the inflection point at approximately 24% r.h. Thus we assumed that multilayer adsorption roughly evolves above 24% r.h. When the amount of water further increases, capillary condensation in the pores is likely to occur [24]. Such capillary condensation may be determined from the steep rise in the adsorption isothermal in the range of 85–100% r.h. The subsequent desorption isotherm deviates at 0% r.h. from the adsorption isotherm, presumably because the measurement time of 40 min per step is not long enough to achieve equilibrium at 30 ${}^{\circ }C$.

### 3.2. Characteristics at Different Levels of Humidity

**Figure 3:**
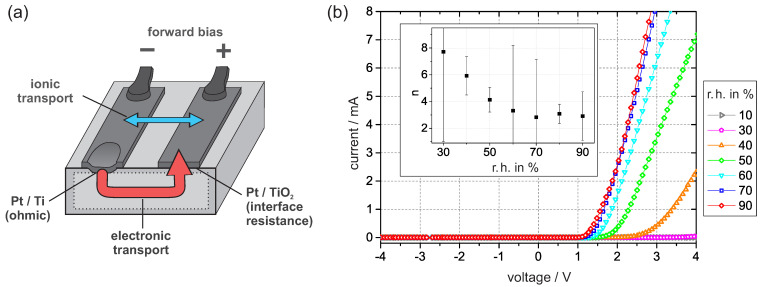
(**a**) The sample setup under the forward bias condition. Schematically shown are the relevant junctions, the electronic transport, and the possible contribution of ionic transport. (**b**) *I–V* curves were recorded at 30 ${}^{\circ }C$ and constant levels of humidity. The inner graph shows the ideality factor *n*, which was obtained by fitting the *I–V* curves according to the concept of an interface-controlled Schottky contact. The error bars are deduced from the fitting standard error.

The sample setup is shown in Figure 3a. The Pt/Ti junction provides low ohmic resistance, while the dominant nonlinear resistance is associated with the Pt/PEO-TiO${}_{2}$ interface. *I–V* curves were recorded at defined levels of humidity in regular time intervals. Initially, when the dry nitrogen flow started, the *I–V* curves began to flatten. After more than 10 h, the current vanished within the resolution of the digital multimeter for applied voltages up to 4 V. As shown in Figure 3b, the intensity of the curve increased again with increasing humidity in the nitrogen flow.

The shape of the *I–V* curves follows the equation for an interface-controlled Schottky junction,

(3a) $$\begin{array}{cc} I(V) & =\left\{A\;A^{*}\;T^{2}\;\exp\left(-\frac{e\Phi_{\mathrm{SB}}}{kT}\right)\right\}\left\{\exp\left(\frac{e(V-IR)}{nkT}\right)-1\right\} \end{array}$$

(3b) $$\begin{array}{cc} I(V) & =I_{S}\left\{\exp\left(\frac{e(V-IR)}{nkT}\right)-1\right\}, \end{array}$$

where *A* is the Schottky contact area, $A^{*}$ is the “Richardson constant”, *T* is the temperature, *e* is the elementary charge, *k* is the Boltzmann constant, $\Phi_{\mathrm{SB}}$ is the Schottky barrier height, *R* is the internal resistance, *n* is the ideality factor, and $I_{S}$ is the saturation current.

By fitting the *I–V* curves according to reference [26], we obtain the ideality factor *n*, which can be used to estimate the density of occupied interface states. According to the values of *n* in Figure 3b, the density of interface states drastically decreases with humidity. This tendency is inverse to the effect of oxygen exposure [6]. To explain our observation, we focus on the electronic charge transport across the Pt/PEO-TiO${}_{2}$ interface.

#### 3.2.1. Interaction of Adsorbed Oxygen and Water

**Figure 4:**
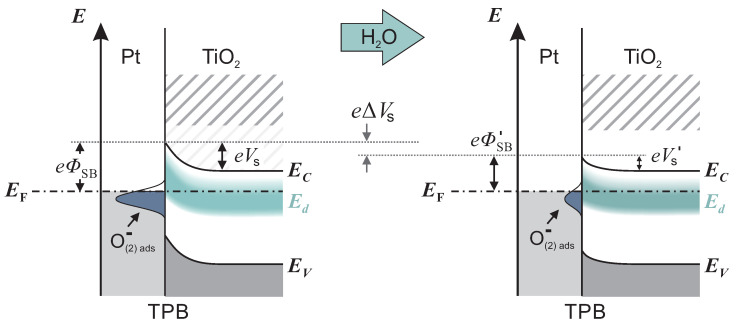
Illustration of the band structure of Pt/TiO${}_{2}$ at three phase boundaries (TPBs). In the left scheme, the depletion and band bending is strongly promoted by the high surface density of ionosorbed oxygen, e.g., ${O^{-}}_{\mathrm{ads}}$. When the humidity increases, the density decreases. The impact on the band structure is shown in the right scheme. In comparison, the band bending is reduced by $e\Delta V_{S}$.

Intrinsic point defects in TiO${}_{2}$, as oxygen vacancies V${}_{O}$ and titanium interstitials Ti${}_{\mathrm{int}}$, induce excess electrons, which can be thermally excited into the conduction band [27]. Therefore, TiO${}_{2}$ with oxygen deficit behaves as a n-type semiconductor. The corresponding electronic states are often summarized in a defect band $E_{d}$, situated approximately 0.1 eV below $E_{C}$ with a maximum density of states appearing 0.9 eV below $E_{F}$ [28]. When a Pt/TiO${}_{2}$ junction is formed, an electron depleted layer within TiO${}_{2}$ evolves due to work function differences between Pt and TiO${}_{2}$. The depletion layer induces a band bending $eV_{S}$, which represents a significant resistance for the electronic transport across the Pt/TiO${}_{2}$ interface. Oxygen ionosorption at three-phase-boundaries (TPBs), i.e., ${O^{-}}_{(2)\;\mathrm{ads}}$/Pt/TiO${}_{2}$, described in Reactions (1) and (2), additionally enhances the depletion layer and increases the band bending by localizing electrons from the occupied conduction band of TiO${}_{2}$ [6]. The left scheme in Figure 4 shows the band bending in an oxygen-containing atmosphere with low humidity, including the defect band $E_{d}$ and oxygen induced acceptor states.

At dry nitrogen or low humidity, e.g., 10% r.h., the current of the *I–V* curve in Figure 3 barely shows any response to the applied voltage, which we attribute to the high interface resistance. The origin of the high resistance is assumed to be oxygen ionosorption from the residual oxygen in the nitrogen, which corresponds to less than 5 $\times 10^{-2}$ Pa. Previous studies on single crystalline TiO${}_{2}$ (rutile phase) showed that oxygen partial pressures in this range already enhance the resistance noticeably [6,9]. The high impact of oxygen, already at low partial pressures, prevents the Pt/PEO-TiO${}_{2}$ material for oxygen sensing in dry atmospheres.

However, in humid atmospheres, the *I–V* curves drastically increase. We attribute the effect to a reaction of H_2_O with ionosorbed atomic oxygen, ${O^{-}}_{\mathrm{ads}}$. When water vapor is provided, water molecules may adsorb associative at a free adsorption site V${}_{\mathrm{ads}}$, summarized in the equilibrium reaction

(4) $$H_{2}O+V_{ads}⇌H_{2}O_{ads}.$$

At TiO${}_{2}$(110), V${}_{\mathrm{ads}}$ is typically a free site at an undercoordinated titanium atom, where water molecules bond with their O end [21]. Atomic ionosorbed oxygen is also situated at these sites. When water is in the immediate vicinity of ${O^{-}}_{\mathrm{ads}}$, water may dissociate by transferring a hydrogen atom to the adjacent ionosorbed oxygen. The formed hydroxyl groups are bound to lattice titanium and are referred to as terminal groups, ${\mathrm{OH}}_{\mathrm{ads}}$. The electron in the reaction

(5) $$H_{2}O+V_{\mathrm{ads}}+{O^{-}}_{\mathrm{ads}}⇌2{\mathrm{OH}}_{\mathrm{ads}}+e^{-}$$

originates from ${O^{-}}_{\mathrm{ads}}$ and is released to the conduction band of TiO${}_{2}$. An analogue reaction for SnO${}_{2}$ is generally accepted, supported by several experiments [29,30,31]. As a consequence of electron injection, the depletion and band bending are reduced. Thus, the resistance decreases and higher current values occur. The change in the band structure is illustrated in Figure 4 by the transition from left to right.

### 3.3. Current Transients at Different Levels of Humidity

**Figure 5:**
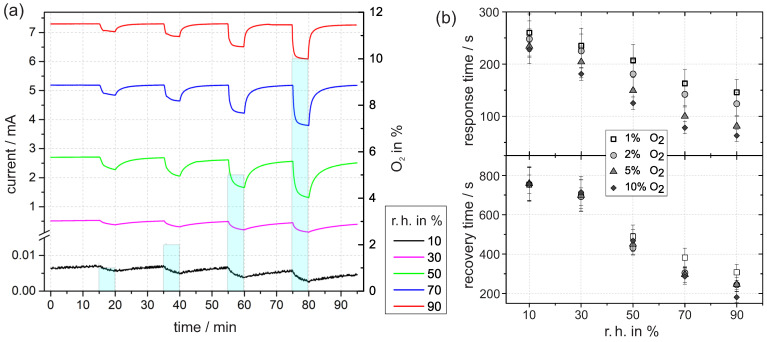
(**a**) Time dependent current at 3 V in constant levels of r.h. at 30 ${}^{\circ }C$ with exposure to different concentrations of oxygen. (**b**) The response time was determined by the time until the signal has fallen to 10% of the initial value relative to the minimum after five minutes of oxygen exposure. In analogous way, the subsequent recovery time was determined by the time until the signal has reached 90% of the following maximum (in nitrogen).

In the following, the results of the time-resolved current measurements with an applied voltage of 3 V are presented. Before, the sample was exposed to a defined r.h. for several hours until the current reaches a constant value. The value can be taken from the first minutes of the transient in Figure 5a. We assume that the constant current corresponds to a quasi-equilibrium concentration of ionosorbed oxygen for the given timescale. With increasing level of humidity, the current value increases, which is therefore attributed to a decrease in the density of ionosorbed oxygen. In accordance to our considerations from Section 3.2.1, this is caused by the replacement of ${O^{-}}_{\mathrm{ads}}$ in Reaction (5). The density of free adsorption sites decreases with increasing humidity because more and more adsorption sites V${}_{\mathrm{ads}}$ are occupied by H_2_O and OH groups. Thus, less sites are accessible for oxygen ionosorption and the equilibrium concentration decreases. As a beneficial side effect for sensing applications, the signal-to-noise ratio increases directly with the current.

#### 3.3.1. Response to Oxygen Exposure

To obtain insights into the kinetics, oxygen at different concentrations is temporally provided. First, the common features of the transients are discussed.

Upon oxygen exposure, each current transient in Figure 5 shows a rapid decrease. The decrease is attributed to the accumulation of ionosorbed oxygen via Reactions (1) and (2).

The absolute current difference of the initial current and the final current after five minutes of oxygen exposure depends on the oxygen concentration (i.e., 1%, 2%, 5%, or 10% O${}_{2}$). The difference increases with higher oxygen levels, which reflects the increased surface density of ionosorbed oxygen species, which rises with the oxygen concentration in the gas flow.

The ambient humidity largely affects the current decay in the transients. The absolute difference between initial value and final value tends to increase with r.h. As before, the difference is mainly reflected by the variable density of ionosorbed oxygen. In nitrogen with low humidity, the density is already relatively high due to free adsorption sites in combination with residual oxygen. When a defined oxygen concentration is added to the nitrogen, the electronic depletion further increases, but the absolute change is small. This is due to the inhibited release of electrons at a high depletion. In contrast to low humidity, the depletion at higher humidity is less pronounced. Therefore, more electrons are accessible and can be transferred to oxygen interface states, which is ultimately reflected in the higher current difference on oxygen exposure.

#### 3.3.2. Oxygen-Induced Changes in Kinetics

An important result from the time-resolved current measurement is that the response and recovery times, shown in Figure 5b, decrease more rapidly with increasing humidity. In case of 5% oxygen exposure, the difference between 10% and 90% r.h. amounts to 153 ± 38 s for the response time and 513 ± 93 s for the recovery time. Together with the improved signal-to-noise ratio in the current, this is a clear indication of the improvement in oxygen sensing with Pt/PEO-TiO${}_{2}$ in the presence of water vapor.

To understand the accelerated kinetics at elevated humidity, we consider which reaction step is rate-determining for accumulation or depletion of ionosorbed oxygen. At high humidity, water layers resemble bulk water, and diffusion of oxygen species through water to the interface occurs, where they adsorb and form acceptor states. If the diffusion process is rate-determining, the response and recovery times at high humidity should be higher compared with low humidity, which was not found. Therefore, we exclude the diffusion process as rate-determining. Instead, we presume that the electron transfer reactions (ETRs) between TiO${}_{2}$ and oxygen adsorbates dominate the overall reaction rate. The rate of ETR is controlled by the band bending at the interface. Figure 4 shows a schematic band model of the TPB at high and low humidity. The band bending $eV_{S}$ at high humidity is significantly less in comparison with the band bending at low humidity. With the reasonable assumption that the ETR rate depends on the band bending, we conclude that the overall kinetic increases with humidity.

In general, the recovery times in the lower half of Figure 5b are significantly larger compared with the response times, which is common for oxygen detection with TiO${}_{2}$ [8,32]. We conclude that the accumulation of ionosorbed oxygen is kinetically faster than the subsequent depletion.

### 3.4. Current Transients at Elevated Temperature

**Figure 6:**
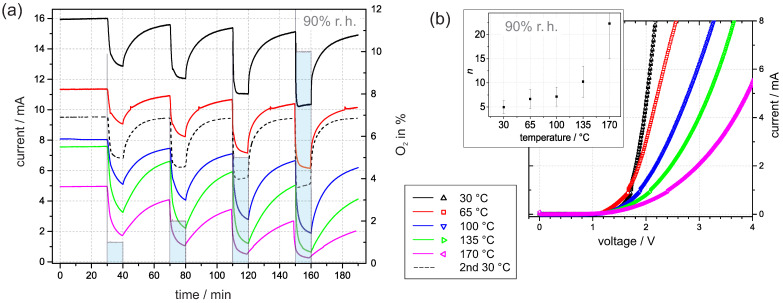
(**a**) Current transients at 3 V in 90% r.h. at temperatures between 30 and 170 ${}^{\circ }C$. The time with oxygen addition was 10 min and the time between oxygen exposure was 30 min. (**b**) The corresponding *I–V* curves were recorded immediately after the transients. The inner graph shows the dependence of the corresponding ideality factor *n* on temperature. The error bars are estimated based on fitting the standard error.

We repeated the time-dependent measurements at 90% r.h. and adjusted the temperature of the sample gradually from 30 to 170 ${}^{\circ }C$. When considering the impact of the temperature, the current expression (3a) for the *I–V* curve predicts an increasing current with increasing temperature. However, the results in Figure 6a reveal a decreasing current with increasing temperature. The temperature dependence can only be adequately addressed if a more detailed picture about the interaction is considered.

In general, an elevated temperature promotes desorption. Hereby, it is generally accepted that water molecules start to desorb at distinctly lower temperatures than adsorbed oxygen species, e.g., temperature-programmed desorption studies on TiO${}_{2}$ rutile suggested that oxygen species, such as ${O^{-}}_{\mathrm{ads}}$ and $O_{2\;\mathrm{ads}}^{-}$, desorb above 600 ${}^{\circ }C$, whereas physically and chemically adsorbed water molecules desorb below 300 ${}^{\circ }C$ [33]. Thus, it is plausible to assume that at room atmosphere, mainly water molecules desorb from Pt/PEO-TiO${}_{2}$ at temperatures up to 170 ${}^{\circ }C$. Hence, water molecules as reaction partners for ionosorbed oxygen in Reaction (5) are missing and the increased density of new adsorption sites facilitates the accumulation of ionosorbed oxygen (from residual oxygen). In conclusion, the absolute current value decreases with increasing temperature. This assumption is supported by the corresponding *I–V* curves in Figure 6b. The embedded graph exhibits a noticeable increase in the corresponding ideality factor *n* with the temperature. This reflects the increasing surface density of ionosorbed oxygen.

After the measurement at 170 ${}^{\circ }C$, the experiment was repeated at 30 ${}^{\circ }C$. Figure 6a shows that the corresponding current at 30 ${}^{\circ }C$ approaches a constant value of around 9.5 mA, and the response to oxygen exposure is repeatable. We conclude from the similarities of the current transients the validity of the proposed model with reversible adsorption and desorption of water. The observed deviation of the absolute value of the current and the drift of the baseline may indicate a change in the oxygen vacancy concentration at elevated temperatures. We suppose that oxygen diffusion into the lattice is promoted with increasing temperature and diminishes the density of oxygen vacancies, which function as electron donators in PEO-TiO${}_{2}$. Lower oxygen vacancy concentration results in decreased conductivity and oxygen lattice diffusion, which explains the baseline drift over time [8].

### 3.5. Donor Effect of Adsorbed Water

Our considerations so far are based on the assumption that the variable density of ionosorbed oxygen species predominantly determines the electronic behavior at the interface. Thereby, we focused on the main mechanism, but we are aware that the oxygen and water interaction on the Pt/PEO-TiO${}_{2}$ surface can be significantly more complex. One of the major contributions stems from a possible donor effect of adsorbed water on Pt/TiO${}_{2}$:

The basic prerequisite is the reaction of water with lattice oxygen. In certain surface configurations on TiO${}_{2}$, water molecules orientate themselves so that one H atom per H${}_{2}$O molecule interacts with a surface oxygen atom [21]. Subsequently, the hydrogen atom is transferred to the adjacent oxygen site, which formally corresponds to the homolytic dissociation of water [34]. As a result, two different hydroxyl groups evolve [33]:

(6) $$H_{2}O+{\mathrm{Ti}}_{\mathrm{Ti}}+O_{O}⇌\left({\mathrm{Ti}}_{\mathrm{Ti}}-\mathrm{OH}\right)+{\left(\mathrm{OH}\right)}_{O}\;.$$

One group is the before-mentioned terminal group, which is bonded at titanium. The other group includes lattice oxygen and is referred to as the rooted group, OH${}_{O}$. At this stage, no ETRs are yet involved. Heiland and Kohl suggested two reactions for SnO${}_{2}$, which extend Reaction (6) by involving an electron donation [35]. In the following, we adapt the reactions for the TiO${}_{2}$ surface. The first reaction respects that the rooted hydroxyl group has a lower electron affinity and can be ionized, expressed in the complete reaction

(7a) $${\left(\mathrm{OH}\right)}_{O}⇌{\left(\mathrm{OH}\right)}_{O}^{+}+e^{-}\;.$$

In the second reaction, a rooted hydroxyl group bonds to a second titanium atom, leaving a vacancy, V${}_{O}$, in place. In the reaction

(7b) $${\mathrm{Ti}}_{\mathrm{Ti}}+{\left(\mathrm{OH}\right)}_{O}⇌\left({\mathrm{Ti}}_{\mathrm{Ti}}-\mathrm{OH}\right)+{V_{O}}^{2+}+2e^{-}$$

the vacancy is already doubly ionized. The corresponding donor states contribute to the intensity of the defect band $E_{d}$. At this point, it should be noted that several studies indicate that rooted OH${}_{O}$ groups also contribute to the defect band $E_{d}$ in the band gap of TiO${}_{2}$(110) [27,36]. Overall, both reactions induce defect states from which electrons can be thermally excited to the conduction band. As a consequence, the depletion and band bending is reduced.

In our current measurement, the reactions with the lattice oxygen, i.e., Reactions (7a) and (7b), could not be distinguished from the replacement Reaction (5). We suppose that Equation (5) prevails when the surface density of ionosorbed oxygen is high. However, when the concentration of ionosorbed oxygen diminishes, Reactions (7a) and (7b) contribute progressively. Findings on SnO${}_{2}$ suggest that the reaction with lattice oxygen is further promoted by platinum additives [31]. We assume that the kinetics of the reactions with lattice oxygen is relatively low at 30 ${}^{\circ }C$. This would explain the waiting time of several hours until a constant current is reached before each measurement.

### 3.6. Ionic Transport along the Surface

So far, we only covered electronic transport through the Pt/PEO-TiO${}_{2}$/Ti structure. For the sake of completeness, we also discuss an ionic contribution. The schematically illustrated sample setup in Figure 5a indicates ionic charge transport along the TiO${}_{2}$ surface between both Pt pads. This is especially conceivable if the specimen is exposed to higher humidity. Hereby, physisorbed water layers on TiO${}_{2}$ occur, which gradually resemble bulk liquid water. Autoprotolysis and charge transport by a Grotthus chain reaction increase the surface conductivity [11]. With additional charge transfer at both platinum electrodes, this would correspond to an electrolytic cell for water splitting. Assuming that the applied voltage exceeds the required decomposition voltage of H${}_{2}$O (including the overvoltages at the Pt electrodes), a nonlinear *I–V* curve is expected (Butler–Volmer equation).

However, two findings contradict such an ionic charge transfer as a predominant origin for the observed *I–V* curves: First, the *I–V* curves should be symmetrical with respect to 0 V. Thus, a non-vanishing current is expected in the positive and negative voltage regions, which could not be confirmed by the *I–V* curves in Figure 3. Second, during electrolysis, hydrogen generation at platinum can be expected. At a current of approximately 7 mA, we would be able to detect traces of molecular hydrogen. This was tested with a thermal conductivity sensor (TCS208F, Gerhard R. Wagner Sensors, Systems & Services, Friedberg, HE, Germany). We found no signal above the TCS baseline for voltages up to 5 V or at any humidity level. We therefore assume that an ionic current contribution was neglectable in our investigation.

## 4. Conclusions

In our study, we demonstrated that adsorbed water improves the oxygen sensing properties of Pt/PEO-TiO${}_{2}$/Ti at moderate temperatures.

Fundamental to our sample design was porous plasma electrolytically oxidized titanium (PEO-TiO${}_{2}$), which was shown to tend toward water multilayer adsorption at elevated humidity. Platinum clusters on PEO-TiO${}_{2}$ function as a catalytically active material and an electrode. The Pt/PEO-TiO${}_{2}$ junction evidently forms a depletion layer, which is additionally controlled by oxygen adsorbates that localize/capture electrons from the TiO${}_{2}$ conduction band. The strong oxygen dependency qualifies for a sensor mechanism, but in room atmosphere, the oxygen partial pressure and thus the interface resistance are so high that hardly any current can be detected for applied voltages up to 4 V. With increasing relative humidity (r.h.) at 30 ${}^{\circ }C$, the current and, ultimately, the signal-to-noise ratio slowly increase. We attribute the effect mainly to the replacement of oxygen adsorbates: A direct reaction with water is proposed, which decreases the surface density of ionized oxygen adsorbates and injects electrons into the depletion zone. In addition, the increasing humidity promotes the occupancy of adsorption sites by H${}_{2}$O or OH groups, resulting in fewer adsorption sites for oxygen adsorbates. In sum, the depletion and ultimately the interface resistance at Pt/PEO-TiO${}_{2}$ decrease. Notably, a further reinforcement by a donor effect of water adsorbates is considered.

Furthermore, our results showed that the response and recovery of the current value on temporary oxygen exposure strongly depends on the ambient humidity. With increasing humidity, the change in the current is accelerated. In the frame of the previous argumentation, we assume that the reduced band bending at higher humidity facilitates electron transfer reactions, which presumably determine the overall reaction rate. Our considerations are further supported by current measurements at elevated temperatures up to 170 ${}^{\circ }C$. The results suggest a predominant effect of oxygen adsorbates with increasing temperature, due to the progressive desorption of water molecules. Thus, the beneficial effects for oxygen sensing in humid atmospheres is limited to low temperatures.

In summary, the study highlights the importance of ambient humidity on the effects of oxygen adsorption on Pt/TiO${}_{2}$. It was shown that elevated humidity can be exploited to counteract the shortcomings of oxygen sensing at low temperatures and high oxygen partial pressures. Our findings expand the current understanding of the complex interplay of water and oxygen on semiconducting metal oxides and suggest ways to benefit from it.

## Data Availability

Not applicable.
